# Potential evidence for transgenerational epigenetic memory in *Arabidopsis thaliana* following spaceflight

**DOI:** 10.1038/s42003-021-02342-4

**Published:** 2021-07-02

**Authors:** Peipei Xu, Haiying Chen, Jinbo Hu, Weiming Cai

**Affiliations:** Laboratory of Photosynthesis and Environment, CAS Center for Excellence in Molecular Plant Sciences, Shanghai Institute of Plant Physiology and Ecology, Chinese Academy of Sciences, Shanghai, China

**Keywords:** Plant molecular biology, Plant stress responses

## Abstract

Plants grown in spaceflight exhibited differential methylation responses and this is important because plants are sessile, they are constantly exposed to a variety of environmental pressures and respond to them in many ways. We previously showed that the *Arabidopsis* genome exhibited lower methylation level after spaceflight for 60 h in orbit. Here, using the offspring of the seedlings grown in microgravity environment in the SJ-10 satellite for 11 days and returned to Earth, we systematically studied the potential effects of spaceflight on DNA methylation, transcriptome, and phenotype in the offspring. Whole-genome methylation analysis in the first generation of offspring (F_1_) showed that, although there was no significant difference in methylation level as had previously been observed in the parent plants, some residual imprints of DNA methylation differences were detected. Combined DNA methylation and RNA-sequencing analysis indicated that expression of many pathways, such as the abscisic acid-activated pathway, protein phosphorylation, and nitrate signaling pathway, etc. were enriched in the F_1_ population. As some phenotypic differences still existed in the F_2_ generation, it was suggested that these epigenetic DNA methylation modifications were partially retained, resulting in phenotypic differences in the offspring. Furthermore, some of the spaceflight-induced heritable differentially methylated regions (DMRs) were retained. Changes in epigenetic modifications caused by spaceflight affected the growth of two future seed generations. Altogether, our research is helpful in better understanding the adaptation mechanism of plants to the spaceflight environment.

## Abstract (web summary)

In order to investigate whether the effects of spaceflight on plants persist in future generations, Xu et al studied the offspring of *Arabidopsis thaliana* seedlings that had been grown in a microgravity environment for 11 days. They found that epigenetic modifications caused by spaceflight potentially affected the growth of two future seed generations, shedding light on the longevity of the effects of spaceflight on plants.

## Introduction

Differences in epigenetic regulation of gene expression induced by environmental stress exposure can persist into the next generation of stressed plants^1^. In some cases, these intergenerational effects can even be passed down to at least two seed generations^2^. In plants, repeated exposure to stress triggers stress adaptation, in which previous stress increases the plant’s ability to respond to future stresses^3^. This concept has been extended to many aspects of the formation of plant stress memories, in which the altered state of plant stress response is transmitted by mitosis or meiosis^1,4,5^. Stress memory in plants is of great interest, including its underlying molecular mechanisms and its potential effects on crop yields, especially in harsh environments. Stress also causes epigenetic changes in DNA methylation profiles and further genomic instability^2^. Previous studies on *Arabidopsis thaliana* and Scots pine (*Pinus silestris*) growing near the Chernobyl reactor have shown that increased genome methylation level is related to genome stability and stress tolerance^6^. Molinier and colleagues reported that exposure to UVC increased the rate of somatic recombination in the plant progeny, whereas increases in UVC-induced homologous recombination frequency (HRF) continued for five generations^1^.

DNA methylation is considered to be a possible mechanism for the formation of stress memories in plants^7^. DNA methylation occurs in three sequence environments in plant genomes, namely mCpG, mCHG, and mCHH, where H is not G, and is thought to play a role in transposable element (TE) silencing and maintenance of genomic stability, and to contribute to gene expression^8^. This potential effect on gene expression suggests that DNA methylation may complement genetic variation and promote phenotypic variation as a means of transmitting genetic information^9^. In fact, the DNA methylation state can be faithfully maintained during mitosis or meiosis by a number of pathways and enzymes. During gametogenesis and spermatogenesis, parental DNA methylation patterns are reconstructed and reproduced, although it is not clear to what extent the patterns of DNA methylation in plant genomes are reprogrammed. Because these processes occur during growth from plant embryos^10^, any cumulative variation in DNA methylation (“epi-alleles”), whether environmentally induced or spontaneous, can persist for generations. DNA methylation shows stable genetic ability and the frequency of epi-alleles in the literature is similar to that of genetic polymorphisms due to spontaneous gene mutations, which may occur at a higher-than-normal frequency under conditions of abiotic stress.

This potential has led to many studies of epigenetic alleles, mediated by intergenerational stress memory, which may open up exciting possibilities for crop (epi-) genomics. However, an alteration in plant phenotype caused by a change in intergenerational methylation is still of a rare occurrence^11^. Due to the lack of early reports on the inheritance of spaceflight effects on plants, in the current study, we systematically studied the potential effects of spaceflight on environmentally induced DNA methylation, using the model plant *Arabidopsis*. With an experiment designed to minimize genetic variation, the ability of plants to create transgenerational stress memories over three successive generations was observed and combined with in-depth DNA methylation profiling and RNA-sequencing analysis. Subsequently, we also tested the possibility of microgravity-induced heritable alleles being associated with adaptive gene expression in spaceflight.

DNA methylation is a major epigenetic modification that modifies gene expression and is passed down at least one generation^7,12^. Previous studies have demonstrated that significant differences in the modification of 5-methylcytosine (5-mC) within the genome of plants undergoing spaceflight, compared with on-ground controls^13,14^. The main purpose of the study was to analyze the transgenerational memory induced by the spaceflight environment in *Arabidopsis thaliana* plants. The experiment was part of the Chinese Academy of Sciences SJ-10 satellite mission and involved a follow-up experiment to the launch of the *Arabidopsis* seedlings which were flown in the SJ-10 recoverable satellite in April^13^. Detailed analysis of the growth and development phenotypes in three consecutive generations from the original spaceflight seedlings was carried out. To evaluate the dynamic DNA methylome alteration and the genetic effects on the offspring following spaceflight, we profiled the genome-wide DNA methylation patterns in the F_1_ generation of *Arabidopsis* seedlings, using whole-genome bisulfite sequencing (WGBS)^15^. Systematic phenotypic observations, combined with genome-wide methylation analysis, revealed a plant adapting comprehensively to the stressful microgravity environment associated with spaceflight, from the perspective of epigenomic methylation level. We report here on how exposure of *Arabidopsis* seedlings to the microgravity environment during spaceflight contributed to the intergenerational changes in plant development and stress response. Some of these changes continued for generations, even in the absence of subsequent stress, and we discuss here how these heritable changes may be due to changes in genomic methylation involved in epigenetic regulation of gene expression.

## Results

### Experimental design of the space vs. ground control comparison treatments, and follow-up experiments

**Fig. 1:**
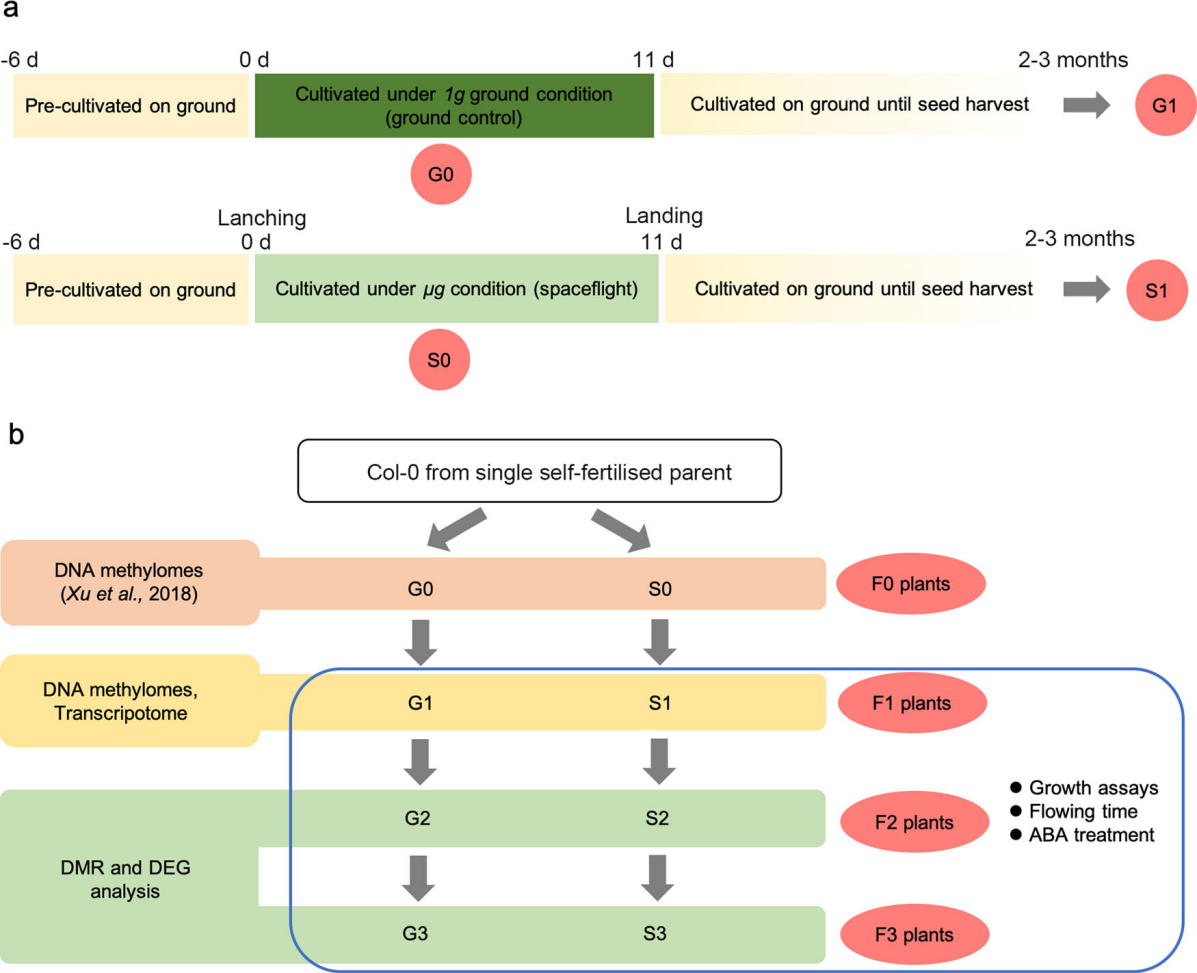
Schematic of experimental procedures on board the SJ-10 recoverable satellite and follow-up experiments. **a** Schematic diagram of *Arabidopsis* seedlings on board the SJ-10 satellite in spaceflight and on the ground, and the follow-up experimental design arrangements. *Arabidopsis seedlings* were transferred from petri dishes 3 d before launching. Seedlings in culture chambers installed in growth chamber unit 1 were grown under microgravity conditions and fixed in space using RNAlater® as described in the previous report^13^. The seedlings in the culture chamber installed in growth chamber unit two in the satellite grew for 11 days under microgravity; after this time of spaceflight, they were still alive, were returned to Earth, and cultivated until seed harvest. **b** Multiple independent lineages began with individual inbred *Arabidopsis* plants, propagated by single-seed descent, with self-fertilization, for three generations, with half of the lineages exposed to the microgravity of space and the remainder maintained on Earth. Intergenerational stress memory tests were performed on the offspring plants. Whole-genome bisulfite sequencing (WGBS) and transcriptome analyses were performed on the F_1_ generation. Various growth phenotypes were assessed in successive F_1_, F_2_, and F_3_ generations.

The Col-0 *Arabidopsis* seedlings were grown in a growth chamber on earth for 6 days before being transferred to one of the two treatments (Fig. 1a, b): either on the Chinese recoverable scientific satellite SJ-10 (S0 population), under microgravity conditions, or on Earth (“ground control”), at 1 *g* gravity load, as the control (G0 population). After 60 h culture, the total DNA was extracted for genome-wide methylation analysis from the G0 and S0 populations of the parent generation. After completion of the spaceflight (11 days), a representative sample of G0 and S0 seedlings was transplanted into soil on Earth to grow them to maturity to measure growth and development traits, and to collect self-fertilized seed from individual plants to breed the next generation (and subsequent generations). This seed production was continued for three more generations, and phenotype analysis was carried out on each generation.

In the F_1_ generation (G1 and S1 populations), DNA was extracted from 6-day-old seedlings of each population and used for genome-wide methylation analysis to detect changes in methylation levels, and to evaluate the genetic and phenotypic effects of DNA methylation at the whole-genome level. Transcriptome sequencing was also performed on the F_1_ generation at the same time. Similar offspring studies were subsequently carried out on later generations of the ground control (G2–G3) and spaceflight groups (S2–S3), all carried out under strict and consistent growth conditions (Fig. 1b).

### The progeny of spaceflight-stressed plants (S1) exhibited different phenotypes, compared with ground controls (G1)

It is known that heavy metal and nutrient deficiency stresses can cause plants to adapt to stress, and, in some cases, this adaptation can be passed on to the offspring of the stressed plants^5,9^. To identify whether intergenerational alterations induced by exposure to microgravity during spaceflight were reflected in plant development and stress tolerance, we systematically compared the offspring of the spaceflight and the ground control populations. Individual spaceflight (S0) and ground control (G0) plants were individually self-fertilized to generate populations of S1 and G1 seeds, and, subsequently S2, S3 and G2, G3 populations (Fig. 1b). The plant growth parameters of the three generations of the two populations, which were assessed included leaf area, root length, flowering time, silique length, root gravitropism, and abscisic acid (ABA)-sensitivity analysis.

**Fig. 2:**
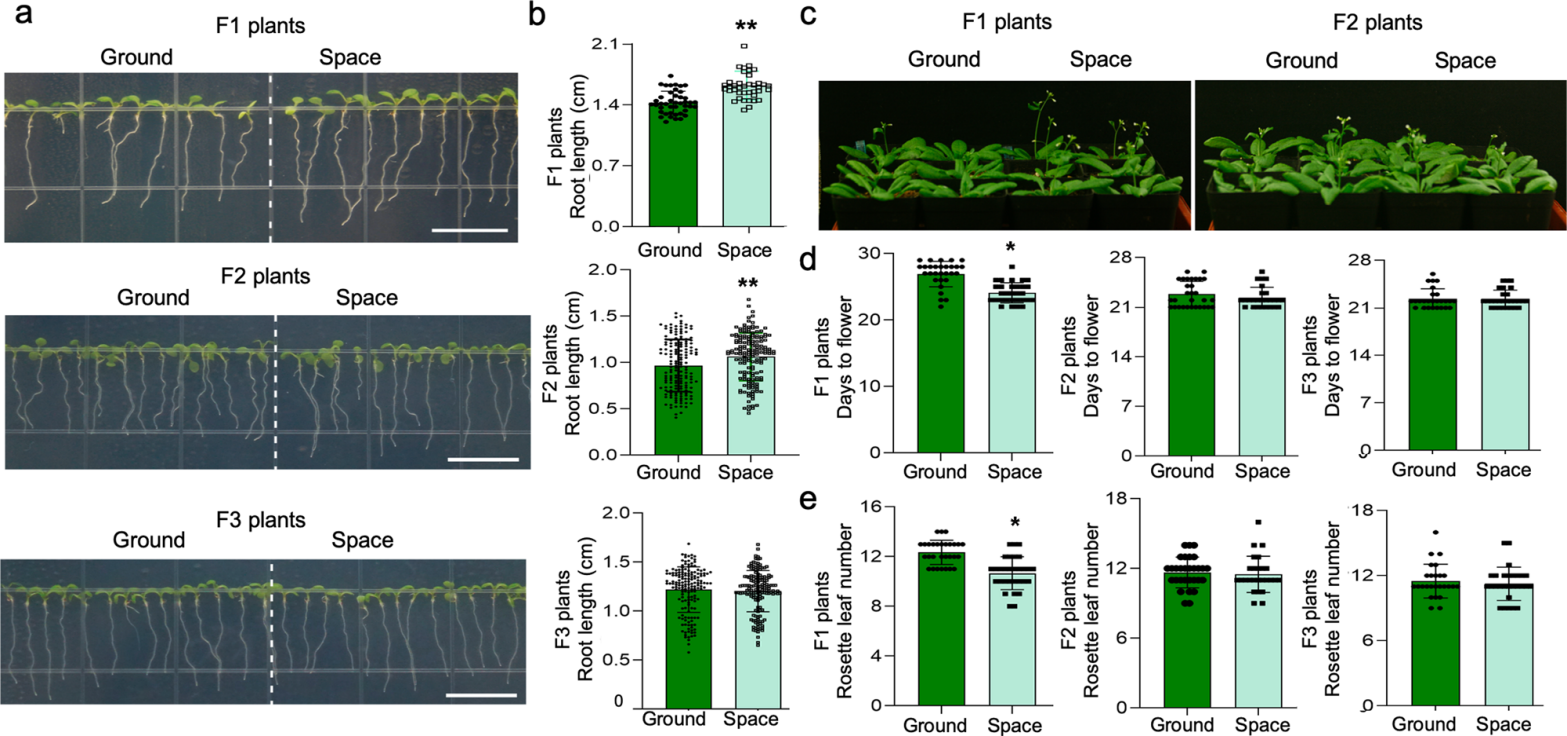
Comparison of the root length, rosette leaf number, and flowering time phenotypes in the offspring of spaceflight and ground control plants. **a**, **b** The length of primary roots of seven-day-old seedlings in the successive F_1_ and F_3_ generations of spaceflight and ground control samples (*n* > 40 for F1 generation, *n* > 150 for F3 generation), and the length of primary roots of six-day-old seedlings in the F_2_ generation (*n* > 160); Scale bar indicates 1 cm. **c**–**e** The flowering time analysis of the three successive F_1_, F_2_, and F_3_ generations of spaceflight and ground control samples (*n* > 30 for each generation). The error bar is the standard deviation. An asterisk *indicates a statistically significant difference between the spaceflight and control samples using Student’s two-tailed *t-*test (*p* < 0.05).

**Fig. 3:**
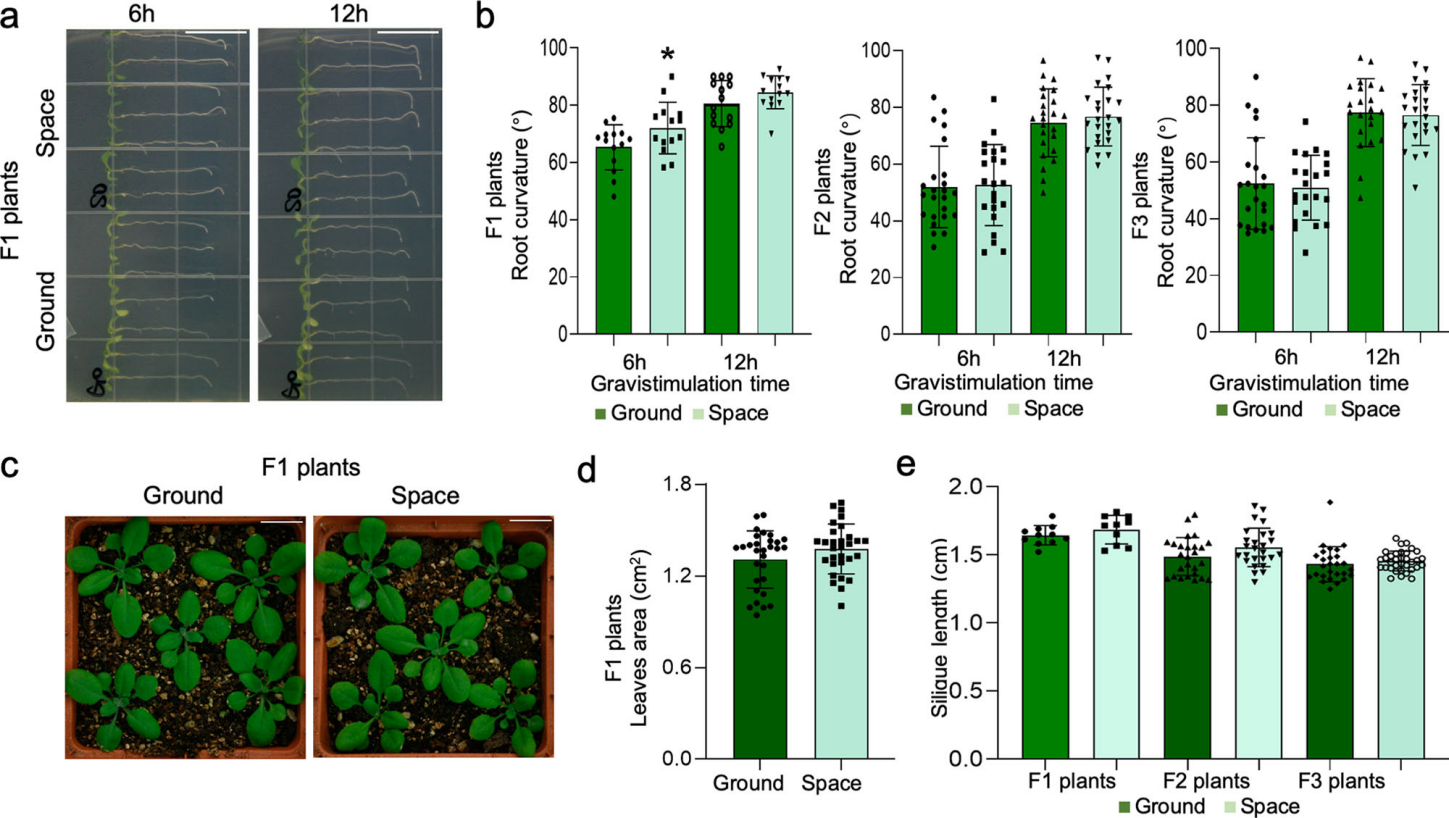
Comparison of the gravitropic orientation analysis, leaf area, and silique length phenotypes in the offspring of spaceflight and the ground control plants. **a**, **b** The 90^o^ change in gravitropic orientation analysis of the offspring seedlings of the spaceflight and ground control samples. The experiment was conducted in the F1 generation and the F2/F3 generations of Arabidopsis seedlings at 8-d-old and 7-d-old, respectively. The data shown are the average values of 14 samples in the F1 generation (*n* = 14) and 24 samples in the F2/F3 generation (*n* = 24). **c**, **d** The rosette leaf area of the offspring of spaceflight and ground control samples. Leaf area was determined by using ImageJ software. The data shown are the mean ± standard deviation values obtained for eight leaves (*n* > 30). **e** The silique length analysis in the offspring of spaceflight and ground samples. The data shown are the mean ± standard deviation values (*n* > 10). An asterisk * indicates a statistically significant difference between the samples by using Student’s two-tailed *t-*test (*p* < 0.05). Scale bar indicates 1 cm.

As we reported earlier, although the leaf area of the S0 *Arabidopsis* seedlings increased significantly in response to spaceflight microgravity treatment, in comparison with the G0 population^13^, there were no significant differences between the offspring populations (Fig. 3c, d). Interestingly, we observed that seedling root length (Fig. 2a, b) and flowering time (Fig. 2c–e) were significantly higher in the spaceflight S1 generation compared with the corresponding ground control generation (G1), but only the effect on the seedling root length phenotype was subsequently preserved in the F_2_ (S2) generation. Furthermore, none of these growth phenotypic differences observed between the S and G F_2_ populations were observed in the F_3_ (S3) generation (Figs. 2, 3).

In terms of ABA sensitivity, the spaceflight F_2_ (S2) generation exhibited significantly higher ABA sensitivity than the control (G2) population, although this advantage could not be observed in the F_3_ generation of the S and G populations (Supplementary Fig. 1). We also observed that the significant difference between S1 and G1 in the phenotype of root curvature after a 90° change in gravitropic orientation was not observed in the F_2_ or F3 generations (Fig. 3a, b). All in all, through our observations of three generations of both populations, we concluded that the phenotypic effects on *Arabidopsis* seedlings of exposure to the stress of the spaceflight environment could be observed in the treated S0 population and their F_1_ offspring, S1, but the heritable effects in subsequent generations varied, with the heritable effect declining with increasing numbers of generations. To our knowledge, our results represent the first systematic analysis of the heritability of microgravity effects of spaceflight on the growth and development of *Arabidopsis*.

### Spaceflight did not cause large-scale changes in genomic DNA methylation patterns, but the distribution of methylation levels changed slightly during the F_1_ generation

**Fig. 4:**
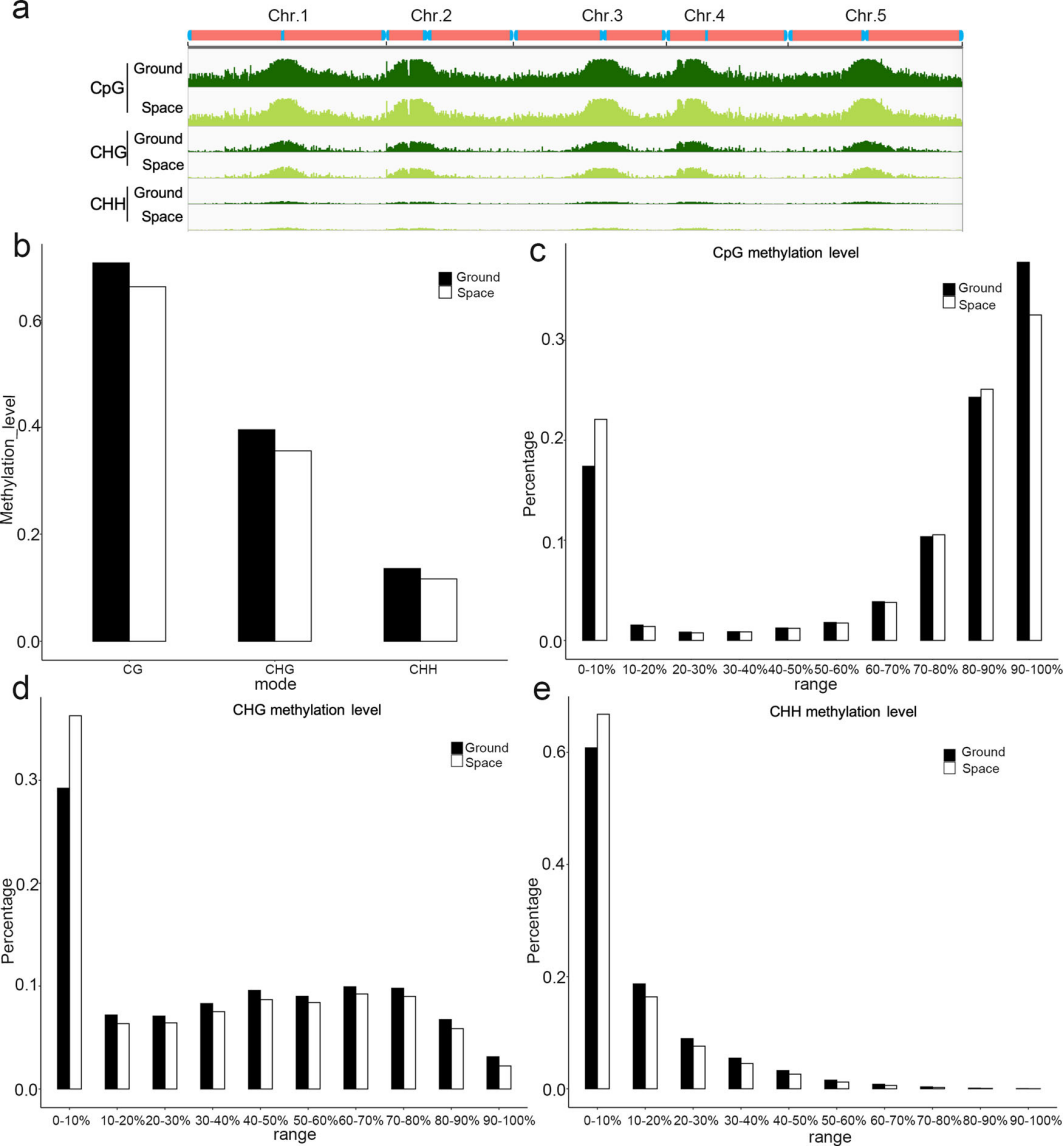
DNA methylation patterns in the *Arabidopsis* genome of the F_1_ generations of the spaceflight and ground control samples. **a** DNA methylation patterns are outlined in the F_1_ generation. The Integrated Genome Viewer (IGV)^53–55^ (http://software.broadinstitute.org/software/igv/) was used to visualize methylation levels of the CpG, CHG, and CHH contexts of chromosomes 1–5 of the offspring of spaceflight and ground control plants. Overview of genome-wide DNA methylation levels in the two samples are shown. **b**–**e** The bar chart lists the overall and proportional methylation of CpG, CHG, and CHH contexts in 10 separate boxes, corresponding to different methylation levels.

The phenotypic changes in the S0 generation, compared with the G0 generation, were associated with major changes in genomic DNA methylation patterns^13^. In order to study the cause of the intergenerational effect of spaceflight on phenotype, we compared the variation in DNA methylation in the F_1_ generation of the two populations (S1 and G1) of *Arabidopsis* (Col-0) seedlings by using the bisulfite-sequencing (BS-Seq) analysis^16^. By assessing the transformation process, the correct cytosine sites for bisulfite transformation were found to be near 98%, indicating that there had been a good conversion and processing rate in the experimental process. Whole-genome bisulfite sequencing (WGBS) revealed genome-wide DNA methylation patterns in the offspring of plants grown in space, as well as their comparable ground controls. The cytosine methylation states of CpG, CHG, and CHH sequences in the F_1_ generations are visualized in the Integrative Genomics Viewer (IGV) (Fig. 4a). Unlike our previous genome-wide DNA methylation data from the parental populations^13^, spaceflight did not cause large-scale changes in DNA methylation in the F_1_ generation, but the distribution of methylation levels was slightly affected by spaceflight. The total methylation level and the distribution of 5-mC in the genome did not change significantly in the genome (Fig. 4a). Figure 4b–e shows the proportional distribution of CpG, CHG, and CHH methylation levels, comparing ground control values with the spaceflight values. In each methylation environment, total methylation decreased slightly during spaceflight, compared with the controls (Fig. 4b), although there was no significant difference in the mean genomic methylation levels between the S1 and G1 samples in each sequence context. However, the level of methylation, at below 10%, was slightly lower in each of the three methylation environments of the ground control population than in the spaceflight population in the F_1_ generation (Fig. 4c–e).

### Spaceflight-induced DNA methylation changes are associated with protein-coding genes in the F_1_ generation

**Fig. 5:**
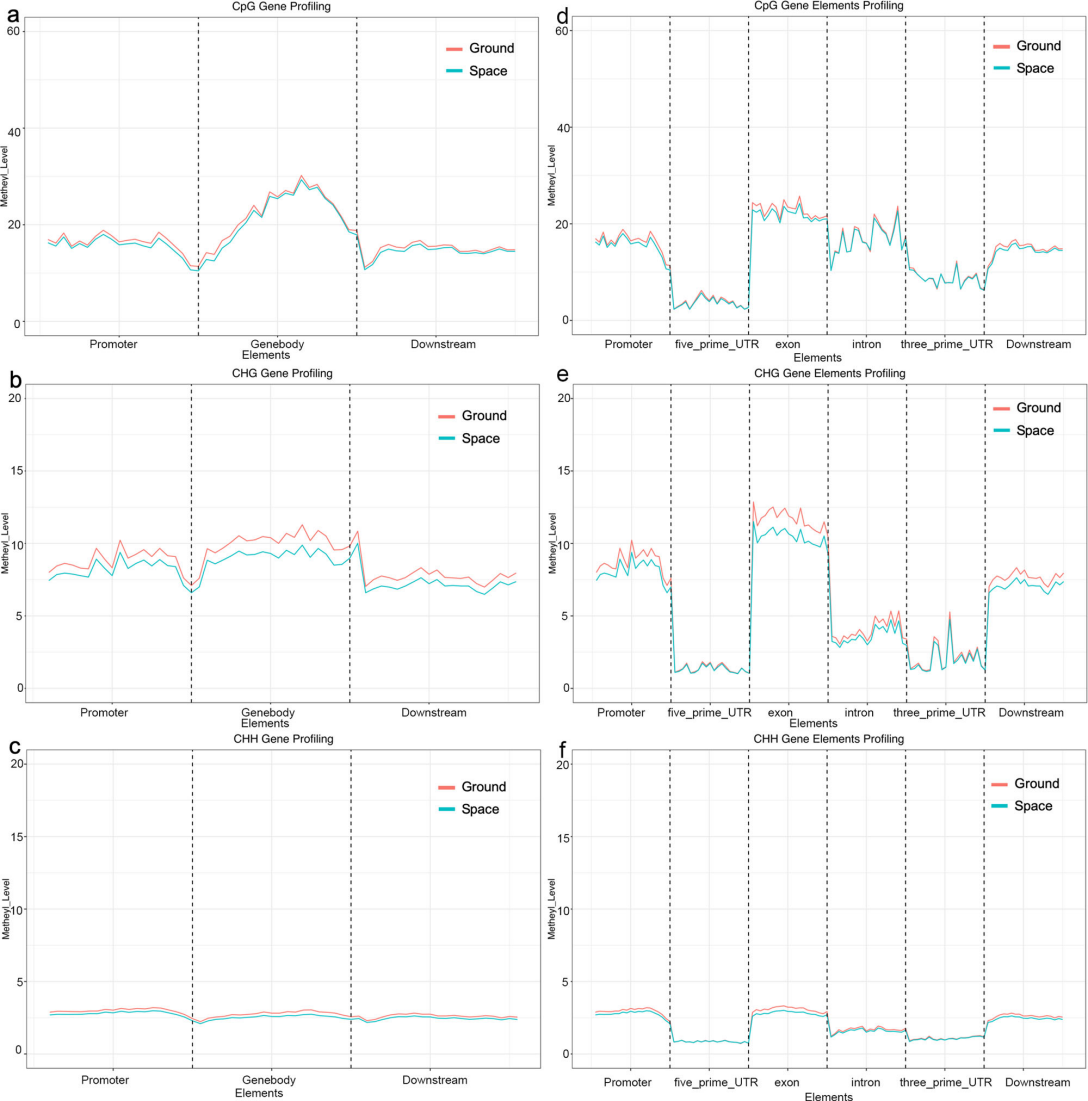
DNA methylation patterns in the *Arabidopsis* genome of the F1 generations of the spaceflight and ground control samples. A line chart showing methylation of the *Arabidopsis* genome in the F_1_ generation of the ground control and spaceflight samples. In **a**, **d** CpG, **b**, **e** CHG, and **c**, **f** CHH environments, including promoters, 5’ untranslated region (UTR), gene bodies, and 3’UTR and distribution of DNA methylation levels in offspring of plants maintained under control and spaceflight conditions. The *x*-axis represents the gene body and its 2-kb upstream and downstream regions. **a**–**c** The *y*-axis represents the average methylation level. **d**–**f** The figure shows three types of methylation patterns, namely CHG, CHH, and CpG.

We further evaluated detailed methylation patterns within genes, including coding sequences (CDS) and noncoding regions. Figure 5 shows the average methylation levels in protein-coding genes in the CpG, CHG, and CHH environments. The results showed that the methylation level at CpG sites was higher than that at CHG and CHH sites in each gene region. In all three environments, the methylation level in the untranslated (UTR) region was lower than that in the coding region (Fig. 5d–f). It was observed that the altered average gene DNA methylation level was largely uniform across the entire gene region. Only in the content of CHG was the methylation level different between the two populations, being slightly lower in the F_1_ generation of the spaceflight population, S1, than in the ground control population, G1 (Fig. 5b, e). Furthermore, no large-scale changes in the average methylation level were detected across the protein-coding region affected by CpG and CHH methylation, and the overall change in DNA methylation was independent of any particular region of the gene being expressed (Fig. 5a, c, d, f).

Differential methylation regions (DMRs) were studied to assess the genome-wide methylation content. The DMRs were determined by comparing the mean methylation levels within a 1000-bp window between the spaceflight and ground control populations and detecting statistically significant methylation levels in the analysis. To further analyze the functional classification of genes with altered methylation patterns, we used Gene Ontology (GO) enrichment to classify these genes^17^. In the biological process category of CpG, altered methylation-related (AMR) genes were enriched for the ammonia assimilation cycle, glutamate biosynthetic processes, protein phosphorylation, protein ubiquitination, epigenetic regulation of gene expression, response to UVC and the tyrosine kinase signaling pathway (Supplementary Fig. 2a). In the biological process category of CHH, the AMR genes were enriched with respect to mitochondrial transport, transmembrane transport processes, and translation processes (Supplementary Fig. 2b). In addition, Kyoto Encyclopedia of Genes and Genomes (KEGG) pathway enrichment analysis was performed for these AMR genes^18^. In the biological process of CpG, AMR genes for mRNA transport, carbon metabolism, amino acid biosynthesis, glycerolipid metabolism, arginine and proline metabolism, and cysteine and methionine metabolism were enriched (Supplementary Fig. 2d). In the CHG context, AMR genes for amino acid biosynthesis, starch metabolism, nucleotide excision repair, the mRNA surveillance pathway and the Fanconi anemia pathway were enriched (Supplementary Fig. 2e).

### The methylation levels of transposable element (TE) genes were modified by spaceflight in the F_1_ generation

**Fig. 6:**
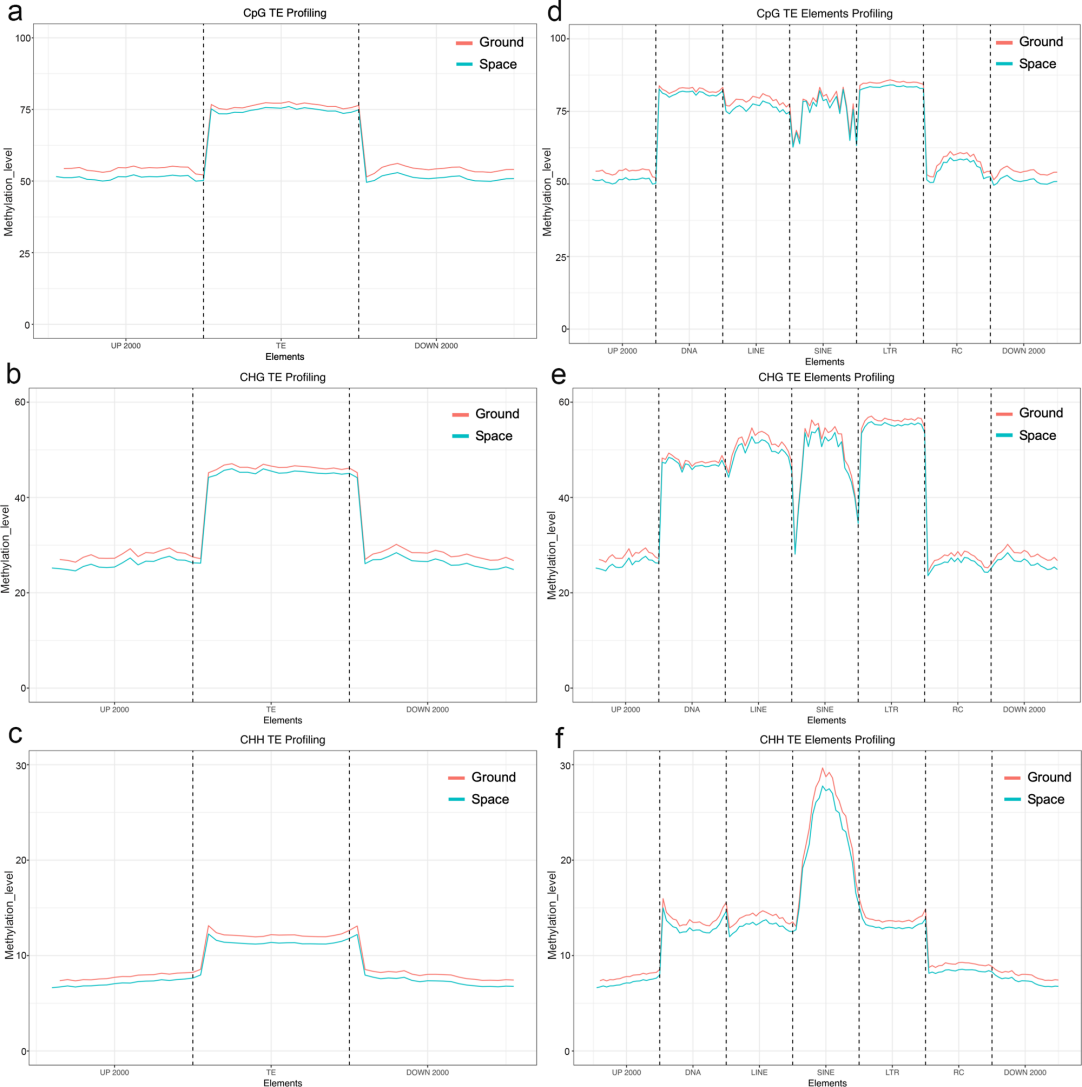
Methylation patterns of different types of transposable elements (TEs) in the *Arabidopsis* genome of the F_1_ generation of the spaceflight and ground control samples. **a**–**c** Methylation pattern of TEs. The figure shows the methylated density distribution of the *Arabidopsis* genome of all TEs in **a** CpG, **b** CHH, and **c** CHG in the F_1_ generation ground control and spaceflight samples. The *x*-axis represents the TE body and its 2-kb upstream or downstream regions. The *y*-axis is the average methylation level. **d**–**f** This study showed the methylation patterns of TEs of five different types, namely DNA, long interspersed nuclear elements (LINE), short interspersed nuclear elements (SINE), long terminal repeats (LTR), and rolling-circle (RC) TEs in the F_1_ generation from ground control and spaceflight plants.

Previous studies have shown that TE mobilization and silencing are often accompanied by the loss of DNA methylation^19^. TEs can affect genome size, produce insertions and other mutations, and affect gene expression patterns^20,21^. Under spaceflight conditions, many different methylated TEs were demethylated in the S0 population^13^. Figure 6a–c shows the methylation patterns of CpG, CHG and CHH contexts in TE regions, as well as the methylation levels in the upstream and downstream regions 2-kb from the CDS. In the spaceflight samples (S1), TEs exhibited a slightly lower methylation level in all three CHG, CHH, and CpG environments (Fig. 6a). These results show that, in these environments, most TEs have different degrees of methylation, and the somewhat hypomethylation of TE sites in the S1 population may be induced by active TEs.

Previous studies had demonstrated that different types of TEs affect methylation levels. In addition, TE methylation levels can affect the expression of transposons under environmental stress^22–24^. In order to further determine the relationship between the methylation state of *A. thaliana* and the various types of TE, TEs were divided into five groups: DNA, long interspersed nuclear elements (LINE), short interspersed nuclear elements (SINE), long terminal repeats (LTR), and rolling-circle (RC) TEs^25,26^. The analysis showed that, compared with other TE types, the methylation signal of RCs was relatively low, whereas SINEs showed significant hypermethylation levels in the CHH context, and all types of TEs in the spaceflight sample showed slightly lower global methylation levels than did those in the ground controls (Fig. 6d–f), indicating that the demethylation mechanism of different types of TEs in the spaceflight samples differed. In addition, we found that the SINEs, LINEs, and LTRs had significantly lower methylation levels in the CHH context than those in the CpG or CHG contexts. In conclusion, the findings suggest that TE demethylation plays a role in adaptation to microgravity.

### Relationship between differential DNA methylation and gene expression

**Fig. 7:**
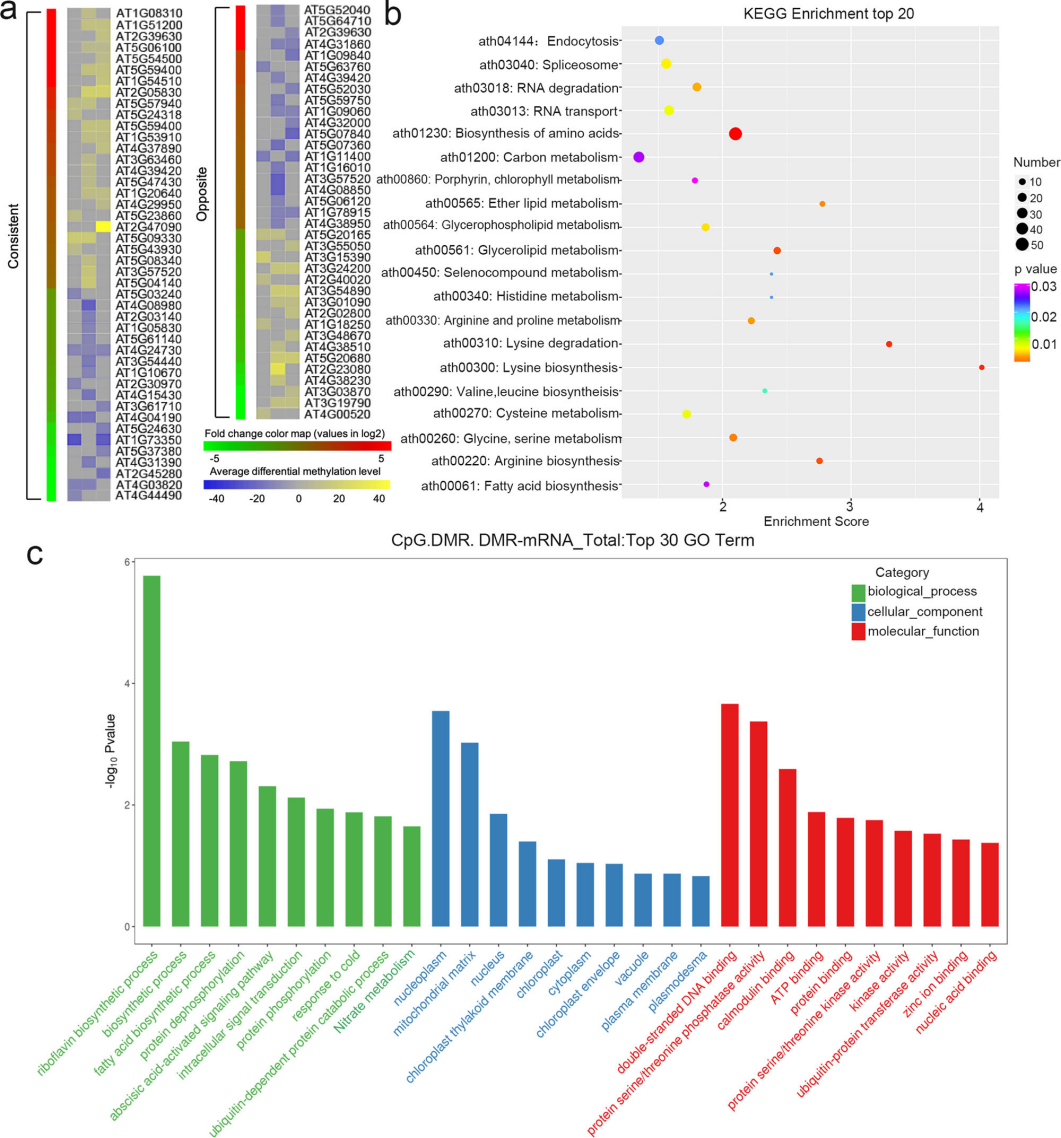
Relationship between differential gene expression and DNA methylation. **a** Heatmap representation of differential gene expression (Log_2_ fold-change) and differential DNA methylation (average of the region); gene body: transcription start site (TSS) to transcription terminations site (TTS), upstream: 2-kb from TSS, downstream: 2-kb from TTS. The opposite trends are indicative of upregulated genes with hypomethylated DMRs (Different Methylation Regions) or downregulated genes with hypermethylated DMRs, whereas a consistent trend shows the converse relationship. **b** KEGG (Kyoto Encyclopedia of Genes and Genomes) pathway and **c** GO (Genetic ontology) enrichment analysis shows a visual representation of spaceflight DMRs and differentially expressed genes (DEGs) that overlapped.

RNA sequencing (RNA-Seq) was used to establish the model of gene expression differences between F_1_ generations of spaceflight (S1) and ground control plants (G1). In the comparison between the spaceflight and ground control populations, about 400 genes were differentially expressed. These genes were associated with responses to abiotic stresses, oxidative stimuli, and hormones, etc. Among all differentially expressed genes (DEGs), 81 differentially expressed genes were also differentially methylated in the CpG context of the S1 population (Fig. 7a). Figure 7a shows the number of DEGs mapped to DMR in the CpG, CHG, and CHH contexts in different gene structures (2-kb upstream and downstream of the transcriptional start site (TSS)). It also provides a complete heatmap of the list of DMR-DEG genes and summarizes the relevant characteristics of this group. For each gene, the ID number was consistent with the differential expression heatmap and indicated the mean differential methylation level of the CpG context in each region. The figure also shows a consistent or opposite trend between differential expression and DNA methylation. The opposite trend occurred for genes, which presented in the gene region as hypomethylated DMR upregulation or CpG-methylated hypermethylated DMR downregulation, while the consistent trend described the inverse relationship. Furthermore, expression of the ABA-activated signaling pathway, the response to abiotic stress stimuli and the cold stimulus, nitrogen compound metabolic process, etc. was examined by Gene Ontology analysis (Fig. 7b). The biosynthesis of amino acids, carbon metabolism, glycolipid metabolism, etc. were examined by KEGG analysis (Fig. 7c). The scatter plots of different gene expressions and the differences of CpG libraries in different gene regions also reflected the relationship between DNA methylation and gene expression of a certain type.

### Isolation and verification of the spaceflight-induced heritable DMRs in the offspring

**Fig. 8:**
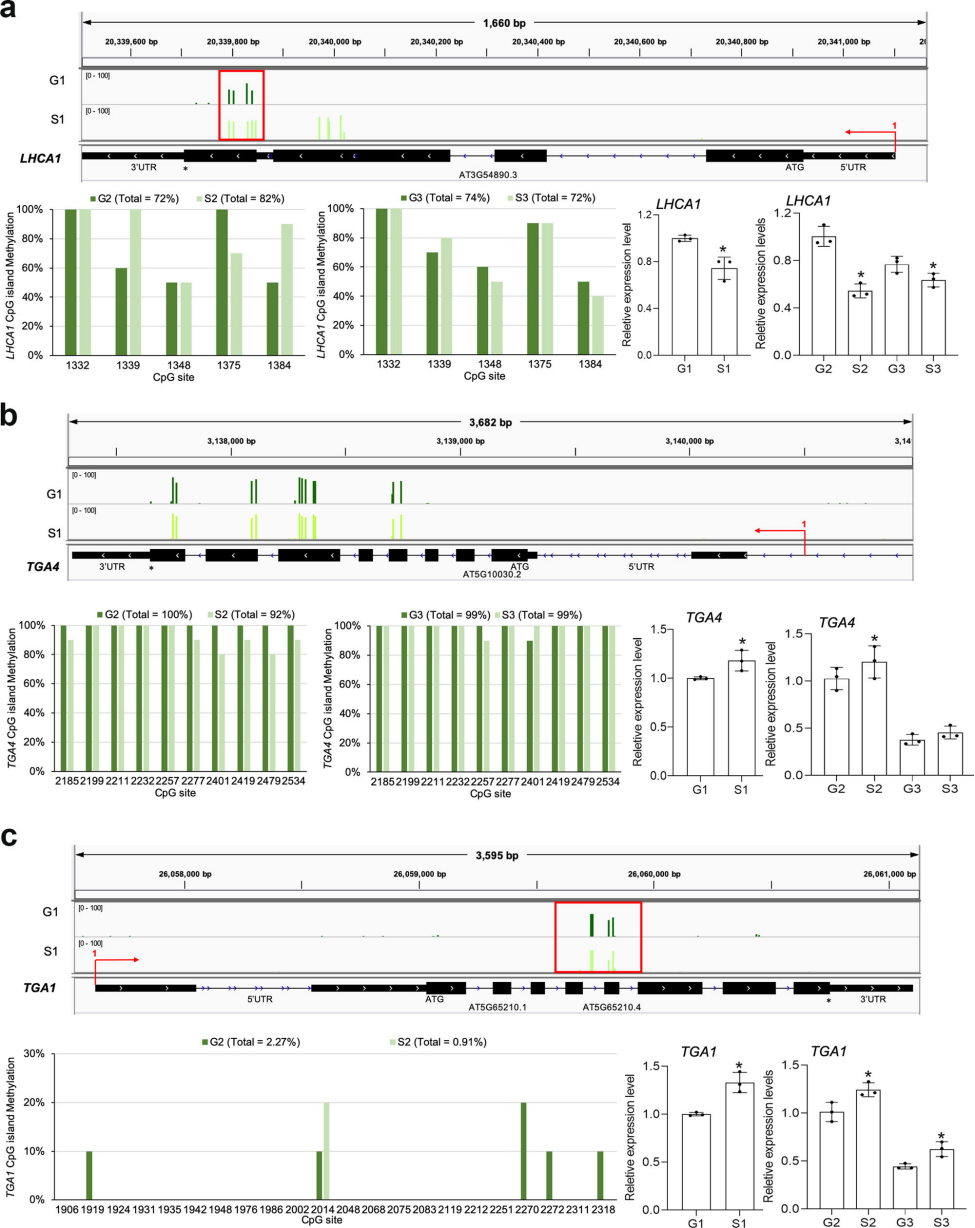
Isolation and verification of the spaceflight-induced heritable DMRs and analysis of the gene expression levels in the offspring. Visualization and BSP (Bisulfite-Sequencing PCR) analysis of selected putative spaceflight-induced heritable DMRs and expression levels of **a** *LHCA1*, **b** *TGA4*, and **c** *TGA1* in the offspring of spaceflight and ground control plants. Visualization of methylation data was performed, using the Integrated Genome Viewer (IGV) (http://software.roadinstitute.org/software/igv). The inherited methylation level of CpG islands was verified by using BSP analysis. The error bar is the standard deviation. An asterisk * indicates a statistically significant difference between the spaceflight and control samples using Student’s two-tailed *t*-test (*p* < 0.05).

Due to the fact that root length, flowering time and silique length were obviously and significantly increased in the spaceflight F_1_ generation (S1), compared with the ground control offspring (G1) (Figs. 2, 3), analysis of the nature of this heritable effect was carried out. The spaceflight-associated DEGs also participated in the photosynthesis pathway, nitrogen compound metabolism and signaling pathway, etc. To further test the relationship between the two aspects, methylation and gene expression, we selected one of the photosynthesis-associated genes, *LHCA1*, and the key nitrate signaling genes, *TGA1/TGA4*, for further analysis. *LHCA1* encodes a component of the light-harvesting complex associated with photosystem I. LHCA1 interacts with WHIRLY1 to change the photochemical activity of photosystem I and participate in the light adaptation of *Arabidopsis*^27^. In accord with the F_1_ generation result, the hypermethylation level of the *LHCA1* gene body in the F_2_ generation was verified by BSP (bisulfite-sequencing PCR) analysis, and this was associated with the corresponding expression level of *LHCA1* being downregulated in the F_1_–F_3_ generations of spaceflight plants, relative to the levels in the corresponding ground control populations (Fig. 8a).

In terms of the nitrogen compound metabolic process and the nitrate signaling pathway, NIN-like transcription factors and *TGA1/TGA4* transcription factors play a central role in nitrate signaling^28–32^. Meanwhile, the spaceflight-associated hypomethylation of the nitrate signaling genes *TGA1* and *TGA4* were identified in the gene body region of the CpG context. We inferred that the increased gene expression level caused by hypomethylation may contribute to the increased nitrogen absorption in the F_1_ generation of the spaceflight offspring (Fig. 8b, c). Furthermore, the hypomethylation levels of the inherited conserved methylation sites in *TGA1* and *TGA4* genes were also identified in the F_2_ generation but not in the F_3_ generation (Fig. 8b, c). To test whether nitrate assimilation was changed, we checked the Nitrate content and NR activity in the F_1_ generation of the spaceflight offspring. We found that spaceflight environment increased Nitrate content and NR activity in the F_1_ generation (Supplementary Fig. S7). Combined with the observation of the growth and development phenotypes (Figs. 2, 3), we inferred that the gene expression changes caused by spaceflight-induced inherited modification of methylation sites could, at least partly, explain the increased growth phenotypes in the spaceflight plant offspring.

Here, we also selected the DMR-DEG genes of the F_1_ generation and showed the distribution of differential methylation patterns by using the IGV browser graphics (Fig. 8). Genes related to the ABA-activated signaling pathway, kinase signaling, and hormone signaling were represented in the DMR-DEG sets. Spaceflight-associated hypomethylation in the gene body region in the CpG context of the ABA-activated pathway genes *CPK26* and *KIN10* occurred in the F_1_ generation. The ABA-activated signaling gene *CPK26* is a member of the calcium-dependent protein kinases (CDPKs), and the Arabidopsis CDPKs are well known for their roles in plant growth regulation and abiotic stress responses^33^. Another gene, *KIN10*, encodes a sucrose nonfermenting 1 (SNF1)-related protein kinase, which is a central integrator of transcription networks in plant stress and energy signaling^34,35^. The methylation levels of the conserved inherited methylation sites of *CPK26* and *KIN10* were detected in the F_2_ and F_3_ generations of the spaceflight plants, but no differences in methylation levels were detected in the selected regions (Supplementary Figs. S3, S4). Altogether, the lower gene expression and the increased ABA sensitivity of *CPK26* and *KIN10* in the F_2_ generation of plants exposed to spaceflight, compared with the ground control plants, may not be due to DNA methylation changes. With respect to the JA-signaling pathway gene, *JAR1*^36^, the *JAR1* gene body was found to be hypomethylated in the spaceflight offspring plants of the F_1_ generation, though this hypomethylation level was not detected in the F_2_ generation (Supplementary Fig. S5). Previous research had demonstrated that the NAC domain transcription factor (TF) VND-INTERACTING1 gene (*VNI1*) was a key regulator of xylem vessel differentiation. The *VNI1* gene body was found to be hypomethylated in the spaceflight plant offspring in the F_1_ generation, although this hypomethylation level was not detected in the F_2_ generation (Supplementary Fig. S6).

## Discussion

As sessile organisms, plants need to constantly adjust their responses to external stimuli in order to adapt to their environment. Due to the fact that seed dispersal is usually quite limited, offspring are likely to be affected by parental growth conditions. Since many environmental pressures last for several generations, other adaptation mechanisms may also exist. Epigenetic modification, which regulates gene expression without altering the DNA sequence, is an attractive alternative mechanism^37^. Epigenetic traits play a key role in controlling gene expression and subsequent responses to the environment^7^. Learning more about plant DNA methylation changes during spaceflight will provide a basic understanding of how plants adapt to stress and, more specifically, to the microgravity environment encountered during spaceflight.

Plants are affected by abiotic and biological environmental factors in many ways. In addition to changes in plant physiology and increased resistance/tolerance responses, genomic dynamics also change. For example, transposable elements are activated by abiotic and biotic stress conditions, mutations are induced by chemical agents, and homologous recombination is enhanced by stresses such as high temperature or UVB^26,38^. However, we know little about the evolutionary impact of these changes; because most of these changes occur in somatic tissues, they cannot be identified in future generations. In plants, germ cells are produced from somatic tissue late in development, so some of the genomic changes acquired during plant growth could be passed on to the next generation. In fact, the frequency of fixed gene mutations increases significantly in the offspring of UVB- or pathogen-exposed plants^38–40^. Genomic changes in the offspring of the stress group are expected to return to original levels. Similar to this general prediction, the reduction of methylation in the F_1_ generation spaceflight samples (S1), compared to the control samples (G1), was obviously lower than that of the parental generation (S0)^13^ (Fig. 4). We show in the current study that the reduction in DNA methylation levels decreased over generations from the original parent plants exposed to the spaceflight environment. The phenotypes observed in successive F_2_ and F_3_ generations are also consistent with this prediction.

Cross-generational effects in genetically uniform populations of plants and animals are usually associated with changes in genomic DNA methylation. This led us to compare the levels of 5-methylcytosine (5-mC) in genomic DNA isolated from the offspring of stressed S0 and non-stressed control G0 plants. The observation of various growth and development phenotypes and stress sensitivity of the offspring plants, cultured under nonstress conditions, was analyzed. Compared with the offspring of the same-generation control plants, the 5-mC content was slightly downregulated in S1 plants grown from seed from plants exposed to spaceflight stress (Fig. 4b), indicating that DNA methylation tended to decrease when stress was not maintained.

In a more detailed study, we measured the overall genomic methylation by WGBS analysis. The different methylation levels between the F_1_ generations of spaceflight and ground samples were compared. Compared with the ground control (G1), the regions with lower methylation levels in S1 were regarded as hypomethylated regions, with these genes being involved in signal transduction, transcription, protein metabolism, and stress response. The general concept of spaceflight-induced DNA methylation changes supports the overall conclusions obtained in an earlier study from ecotype Col-0 seedlings growing on spaceflight^13^; however, previous research by Zhou et al. (2019) did not observe wide-scale hypomethylation during spaceflight, in a study with the *Arabidopsis* ecotype Ws-0, that was germinated and lived on the International Space Station, ISS^14^. We speculate that the inconsistent results were mainly due to the ISS experiment and spaceflight experiment itself or the use of different *Arabidopsis* ecotypes. Recent transcriptome sequencing studies have shown that gene expression profiles and alternative gene splicing patterns in *Arabidopsis* ecotypes Col-0 and Ws-0 differed significantly in their adaptation to microgravity^41,42^, and these results suggest that the two ecotypes may have different genomic methylation patterns in response to the microgravity environment. Other possible reasons, such as the growth hardware, and the age of the seedlings on the spaceflight, may also play a role. This research experiment also highlights the importance of natural variation impacts when designing and interpreting changes related to the space microgravity environment. Of course, we should acknowledge the limitations of spaceflight experiments themselves. We cannot resolve gravity affects without performing a control grown on a centrifuge in space in the same hardware, and seedlings were only exposed to spaceflight for a short period of time, so it was not known how this translates to long-term growth of seedlings.

Similar to our result, previous research reported an increase in the plant root length in spaceflight and simulated microgravity (Random Positioning Machine, RPM) experiments^43^. They also found the red light was able to compensate for deleterious effects of microgravity on the root cell growth^43^. In addition, cell cycle acceleration and changes in essential nuclear functions induced by simulated microgravity in a synchronized *Arabidopsis* cell culture were also observed; as well as some observations on changes in chromatin-based regulation increased chromatin condensation, and the overall depletion of nuclear transcription^44^. It should be noted that this work shows that microgravity promotes genomic methylation levels, a conclusion that is inconsistent with our results. One reason, we speculate, is that spaceflight experiments and simulated microgravity experiments are different in themselves, or that the cultured cells and plant seedlings do not respond exactly the same way to microgravity stimulation. Another study showed that gravitational and magnetic field variations synergized to cause subtle variations in the global transcriptional state of *Arabidopsis* in vitro callus cultures^45^. These results expand our knowledge of how culture plant cells and plant seedlings are affected by microgravity.

Interestingly, we observed that root length, flowering time, and silique length were significantly increased in the spaceflight F_1_ generation compared with the ground control F_1_ offspring, but that only the root length phenotype was preserved in the F_2_ generation (Figs. 2, 3). Here, we also detected transcriptome changes in the F_1_ generation plants, using RNA-Seq analysis, and we identified many gene expression changes between the S1 generation of spaceflight-stressed plants and the G1 ground control plants. Three independent repeat sequences were used to compare the transcriptome of S1 and G1. Expression of 206 genes was upregulated at the RNA level and that of 188 genes was downregulated, when using 2-fold changes and *p* < 0.05 differences in expression as threshold criteria. This DEG group contained many genes involved in abiotic stress response and signaling. We identified spaceflight-associated hypomethylation of nitrate signaling genes *TGA1* and *TGA4* in the gene body region of the CpG context, which may contribute to the increased nitrogen absorption efficiency observed in the F_1_ generation of spaceflight offspring (Fig. 8b, c). Furthermore, the hypomethylation level of *TGA1* and *TGA4* inherited conserved methylation sites were also identified in the F_2_ (S2) generation but not in the F_3_ (S3) generation (Fig. 8b, c). We inferred that the gene expression changes caused by microgravity-induced inherited modification of methylation sites could explain, at least partly, the increased growth phenotype in the spaceflight plant offspring.

In *Arabidopsis*, CpG methylation is mainly controlled by the methyltransferase, MET1, and the chromatin remodeling agent, decreased DNA methylation1 (DDM1). DNA methylation is also associated with genomic stability, especially with respect to transposable factors. We revealed here the relationship between DNA methylation and gene expression, which was involved in many types of abiotic stress genes previously associated with spaceflight responses, including stress response, cold response, cell wall remodeling, nitrate metabolism, plant hormone, Ca^2+^ signaling, etc. In the offspring, we focused on the validation and analysis of ABA-responsive kinase signaling genes and nitrogen metabolism genes, the results showing that the spaceflight-induced genome methylation alterations, which were passed down to their offspring, contributed to the changes in gene expression level between the S and G samples, which result in the transgenerational adaptation by *Arabidopsis* to the spaceflight environment. At the same time, the spaceflight-induced DNA methylation difference between S0 and G0 tended to decrease between generations. All in all, our results are helpful in improving the strategies by which plants adapt to stress, and, more importantly, to the outer space environment.

## Methods

### Plant preparation and growth conditions

The experiment is part of the SJ-10 spaceflight project, which was launched from the Jiuquan satellite launch center in April 2016. Sample preparation prior to launch were consistent with launch conditions described in the previous article^13^. The scientific equipment used in our spaceflight study was obtained from Shanghai Institution of Technical Physics. The specifications of the equipment box, plant growth parameters, and SJ-10 satellite flight parameters are described in the previous reports^13,46,47^. According to our experimental design, in one part of the experiment, the seedlings continued to grow for 60 h under microgravity conditions. In another part, the seedlings grew in microgravity for 11 days in culture unit 2 and were still growing when they returned to Earth. Under spaceflight and ground conditions, all seedlings were grown in a growth chamber under 8000 lx, 22 ± 2 °C and a 16-h photoperiod. The g-profile during launch was as follows: the first-level maximum static overload was 4.8 g in flight for 150 s; the second-level maximum static overload was 6.0 g in flight for 180 s^13^. Micro vibration level is superior to 1.5 mg for long-term, 7 mg for transient peak. Quasi steady state microgravity influenced by attitude adjustment thruster: 10^−6^–10^−5^ g^48,49^. The ground controls were cultivated in a growth chamber, and grown for 11 days on Earth under the similar conditions as the spaceflight samples. It should be noted that the plant experimental unit is airtight, sealed on the ground and sent to the space. So the gas composition in the plant culture unit was the same as on-ground control.

### Flowering time analysis

Flowering time was measured by observing the time from germination to when the first flower bud on each *A. thaliana* plant opened, and counting the number of rosette leaves.

### ABA sensitivity assay

ABA treatment was performed at the germination stage by using the 1/2 Murashige & Skoog (MS) medium containing 0, 0.5, or 2 μM ABA. The plants were photographed after treatment for the times indicated.

### Root gravitropism assay

Four-day-old seedlings grown on a Petri dish were incubated horizontally for a period of time to calculate the angle of root curvature, using the ImageJ software (https://imagej.net).

### Total RNA and DNA extraction

Arabidopsis seedlings grow on plates. Each plate represents a biological replicate of 11-day-old seedlings, consisting of 15–18 individual seedlings for genomic DNA extraction and the remainder (9–12 seedlings) for RNA extraction. RNA was extracted using the RNeasy Plant Mini Kit (Qiagen, Hilden, Germany), and the quality and quantity of RNA samples were evaluated using an Agilent 2100 Bioanalyzer (Agilent Technologies, Inc.). Total DNA was extracted using the methodology described in previous reports^13^. Three biological replicates were used from the spaceflight and ground control samples for each of RNA-Seq and WGBS analysis.

### RNA sequencing (RNA-Seq) and whole-genome bisulfite DNA sequencing

RNA was extracted and measured according to the manufacturer’s guidelines. The RNA-Seq library and genome-wide bisulfite sequencing was carried out as described in the previous reports^13,14^. An Illumina sequencing library was constructed using NEBNext Ultra DNA Illumina Prep Kit (New England Biolabs, Ipswich, MA, USA), in combination with Illumina-specific methylation and barcode adapters. The EZ DNA Methylation™ Kit (Zymo Research, Irvine, CA, USA) was used to convert the library to bisulfite, according to the manufacturer’s protocols. The nine barcoded bisulfite-Seq libraries and nine barcoded RNA-Seq libraries (with three biological replicates for each sample) were sequenced on a HiSeq3000 instrument. The primers used are shown in Supplementary Tables. S1,S2.

### Read alignment and processing

FastQC was used to evaluate the quality of RNA-Seq sequence data. Low-quality sequences, such as adapters, were removed. Gene expression was performed by using the RSEM software (http://deweylab.github.io/RSEM/). The expected read counts per kilobase of transcripts per million map reads and fragments were extracted for further analysis. The threshold value of significantly differentially expressed genes was set at a false discovery rate (FDR) of 0.05, and a fold change was greater than 2. The input from Bisulfite-Seq was fine-tuned and quality control was carried out. Reads were compared with the reference genome of *A. thaliana* (Col-0) using BSmap^50^. The differential methylation region (DMR) was identified using the previously described method^13^. DMR was classified according to the characteristics of the gene body, promoter and downstream region. The ratio of cytosine transformed in CG, CHG, and CHH environments was calculated using reads labeled with the reference genome, indicating the conversion efficiency of bisulfite.

### PCR program for bisulfite sequencing

Samples were pretreated with the QIAGEN® EpiTect Bisulfite Kit, and the Genemark® Plant Genomic DNA Purification Kit was used to extract genomic DNA. The PCR program settings for cloning fragments were as described in the previous report^13^.

### Gene ontology analysis

Genetic ontology (GO) annotations were performed using agriGO v2.0^51,52^ (http:://systemsbiology.cau.edu.cn/agriGOv2/index.php), a public online tool and database. The significance level for GO terms was FDR < 0.05.

### Kyoto Encyclopedia of Genes and Genomes (KEGG) pathway enrichment analysis

KEGG (http://www.genome.jp/kegg/) analysis was used to determine the pathway enrichment of functional DMRs or differential expression genes with *p*-values less than 0.05.

### Analysis of transposable elements (TEs)

A repeat masking program (http://www.repeatmasker.org/) was used to separate and analyze TEs. We used Fisher’s exact tests to study TEs for changes in methylation levels. The FDR method was used multiple times to test adjustments. A *p-*value less than 0.05 was defined as a methylated TEs with significant changes.

### Statistics and reproducibility

An asterisk * indicates a statistically significant difference between the samples by using Student’s two-tailed *t*-test (*p* < 0.05). Sample sizes are indicated in detail in each figure caption, main text, or corresponding method sessions.

### Reporting summary

Further information on research design is available in the Nature Research Reporting Summary linked to this article.

## Supplementary information

Supplementary Information

Supplementary Data 1

Reporting Summary

## Data Availability

The data that support this study are available within the article and its Supplementary Information files or available from the authors upon request. Source data are available as Supplementary Data 1. The WGBS and RNA-Seq source data reported in this paper are available in GEO under number SUB6407289.

## References

[1] Molinier J, Ries G, Zipfel C, Hohn B (2006). Transgeneration memory of stress in plants. Nature.

[2] Cong, W. X. et al. Transgenerational memory of gene expression changes induced by heavy metal stress in rice (Oryza sativa L.). *BMC Plant Biol*. **19**, 282 (2019). 10.1186/s12870-019-1887-7 PMC6598230 31248374

[3] Rahavi MR, Migicovsky Z, Titov V, Kovalchuk I (2011). Transgenerational adaptation to heavy metal salts in Arabidopsis. Front. Plant Sci..

[4] Mirouze M, Paszkowski J (2011). Epigenetic contribution to stress adaptation in plants. Curr. Opin. Plant Biol..

[5] Murgia, I. et al. Analysis of the transgenerational iron deficiency stress memory in Arabidopsis thaliana plants. *Front. Plant Sci*. **6**, 745 (2015). 10.3389/fpls.2015.00745 PMC4585125 26442058

[6] Kovalchuk O (2003). Genome hypermethylation in Pinus silvestris of Chernobyl—a mechanism for radiation adaptation?. Mutat. Res. Fund. Mol. Muta..

[7] Chinnusamy V, Zhu JK (2009). Epigenetic regulation of stress responses in plants. Curr. Opin. Plant Biol..

[8] Richards EJ (1997). DNA methylation and plant development. Trends Genet.

[9] Thiebaut F, Hemerly AS, Ferreira PCG (2019). A role for epigenetic regulation in the Adaptation and stress responses of non-model plants. Front. Plant Sci..

[10] Boavida LC, Vieira AM, Becker JD, Feijo JA (2005). Gametophyte interaction and sexual reproduction: how plants make a zygote. Int. J. Dev. Biol..

[11] Ganguly DR, Crisp PA, Eichten SR, Pogson BJ (2017). The Arabidopsis DNA methylome is stable under transgenerational drought stress. Plant Physiol..

[12] Ueda, M. & Seki, M. Histone modifications form epigenetic regulatory networks to regulate abiotic stress response. *Plant Physiol.* **182**, 15–26 (2019) 10.1104/pp.19.00988 PMC6945856 31685643

[13] Xu P, Chen H, Jin J, Cai W (2018). Single-base resolution methylome analysis shows epigenetic changes in Arabidopsis seedlings exposed to microgravity spaceflight conditions on board the SJ-10 recoverable satellite. npj Microgravity.

[14] Zhou M, Sng NJ, LeFrois CE, Paul AL, Ferl RJ (2019). Epigenomics in an extraterrestrial environment: organ-specific alteration of DNA methylation and gene expression elicited by spaceflight in Arabidopsis thaliana. BMC Genomics.

[15] Cokus SJ (2008). Shotgun bisulphite sequencing of the Arabidopsis genome reveals DNA methylation patterning. Nature.

[16] Rackham OJ, Dellaportas P, Petretto E, Bottolo L (2015). WGBSSuite: simulating whole-genome bisulphite sequencing data and benchmarking differential DNA methylation analysis tools. Bioinformatics.

[17] Zhou X, Su Z (2007). EasyGO: Gene Ontology-based annotation and functional enrichment analysis tool for agronomical species. BMC Genomics.

[18] Mao X, Cai T, Olyarchuk JG, Wei L (2005). Automated genome annotation and pathway identification using the KEGG Orthology (KO) as a controlled vocabulary. Bioinformatics.

[19] Orlowska R, Machczynska J, Oleszczuk S, Zimny J, Bednarek PT (2016). DNA methylation changes and TE activity induced in tissue cultures of barley (Hordeum vulgare L.). J. Biol. Res. (Thessalon.).

[20] Martienssen R, Transposons DNA (1998). methylation and gene control. Trends Genet.

[21] Fedoroff N (2000). Transposons and genome evolution in plants. Proc. Natl Acad. Sci. USA.

[22] Laurie JD, Linning R, Wong P, Bakkeren G (2013). Do TE activity and counteracting genome defenses, RNAi and methylation, shape the sex lives of smut fungi?. Plant Signal. Behav..

[23] Migicovsky Z, Kovalchuk I (2014). Transgenerational changes in plant physiology and in transposon expression in response to UV-C stress in Arabidopsis thaliana. Plant Signal. Behav..

[24] Ibarra CA (2012). Active DNA demethylation in plant companion cells reinforces transposon methylation in gametes. Science.

[25] Xing MQ (2015). Global analysis reveals the crucial roles of DNA methylation during rice seed development. Plant Physiol..

[26] Joly-Lopez Z, Bureau TE (2014). Diversity and evolution of transposable elements in Arabidopsis. Chromosome Res..

[27] Huang, D. M., Lin, W. F., Deng, B., Ren, Y. J. & Miao, Y. Dual-located WHIRLY1 interacting with LHCA1 alters photochemical activities of photosystem I and is involved in light adaptation in Arabidopsis. *Int. J. Mol. Sci*. **18**, 11 (2017). 10.3390/ijms18112352 PMC5713321 29112140

[28] Alvarez JM (2014). Systems approach identifies TGA1 and TGA4 transcription factors as important regulatory components of the nitrate response of Arabidopsis thaliana roots. Plant J..

[29] Canales J, Contreras-Lopez O, Alvarez JM, Gutierrez RA (2017). Nitrate induction of root hair density is mediated by TGA1/TGA4 and CPC transcription factors in Arabidopsis thaliana. Plant J..

[30] Mu X, Luo J (2019). Evolutionary analyses of NIN-like proteins in plants and their roles in nitrate signaling. Cell Mol. Life Sci..

[31] Konishi M, Yanagisawa S (2013). Arabidopsis NIN-like transcription factors have a central role in nitrate signalling. Nat. Commun..

[32] Marchive, C. et al. Nuclear retention of the transcription factor NLP7 orchestrates the early response to nitrate in plants. *Nat. Commun.* **4**, 1713 (2013). 10.1038/ncomms2650 23591880

[33] Arimura G, Maffei ME (2010). Calcium and secondary CPK signaling in plants in response to herbivore attack. Biochem. Biophys. Res. Commun..

[34] Tahtiharju S, Sangwan V, Monroy AF, Dhindsa RS, Borg M (1997). The induction of kin genes in cold-acclimating Arabidopsis thaliana. Evidence of a role for calcium. Planta.

[35] Simon NML, Sawkins E, Dodd AN (2018). Involvement of the SnRK1 subunit KIN10 in sucrose-induced hypocotyl elongation. Plant Signal. Behav..

[36] Suza WP, Staswick PE (2008). The role of JAR1 in Jasmonoyl-L: -isoleucine production during Arabidopsis wound response. Planta.

[37] Zhang M, Kimatu JN, Xu K, Liu B (2010). DNA cytosine methylation in plant development. J. Genet. Genomics.

[38] Gu X, Nylander E, Coates PJ, Fahraeus R, Nylander K (2015). Correlation between reversal of DNA methylation and clinical symptoms in psoriatic epidermis following narrow-band UVB phototherapy. J. Invest. Dermatol..

[39] Boyko A (2007). Transgenerational changes in the genome stability and methylation in pathogen-infected plants: (virus-induced plant genome instability). Nucleic Acids Res..

[40] Zhang X (2012). Dynamic differential methylation facilitates pathogen stress response in Arabidopsis. Proc. Natl Acad. Sci. USA.

[41] Choi WG, Barker RJ, Kim SH, Swanson SJ, Gilroy S (2019). Variation in the transcriptome of different ecotypes of Arabidopsis thaliana reveals signatures of oxidative stress in plant responses to spaceflight. Am. J. Bot..

[42] Beisel NS, Noble J, Barbazuk WB, Paul AL, Ferl RJ (2019). Spaceflight-induced alternative splicing during seedling development in Arabidopsis thaliana. npj Microgravity.

[43] Valbuena MA (2018). The combined effects of real or simulated microgravity and red-light photoactivation on plant root meristematic cells. Planta.

[44] Kamal KY, Herranz R, van Loon J, Medina FJ (2019). Cell cycle acceleration and changes in essential nuclear functions induced by simulated microgravity in a synchronized Arabidopsis cell culture. Plant Cell Environ..

[45] Manzano AI (2012). Gravitational and magnetic field variations synergize to cause subtle variations in the global transcriptional state of Arabidopsis in vitro callus cultures. BMC Genomics.

[46] Yu J (2019). Homogeneous InGaSb crystal grown under microgravity using Chinese recovery satellite SJ-10. npj Microgravity.

[47] Li N (2018). Microgravity-induced alterations of inflammation-related mechanotransduction in endothelial cells on board SJ-10 satellite. Front. Physiol..

[48] Hu WR (2014). Space program SJ-10 of microgravity research. Microgravity Sci. Tech..

[49] Wang Y (2016). Establishing and evaluation of the microgravity level in the SJ-10 recoverable satellite. Aerosp. China.

[50] Li, Y. X. & Li, W. BSMAP: whole genome bisulfite sequence MAPping program. *BMC Bioinformatics* **10**, 232 (2009). 10.1186/1471-2105-10-232 PMC2724425 19635165

[51] Du Z, Zhou X, Ling Y, Zhang Z, Su Z (2010). agriGO: a GO analysis toolkit for the agricultural community. Nucleic Acids Res..

[52] Tian T (2017). agriGO v2.0: a GO analysis toolkit for the agricultural community, 2017 update. Nucleic Acids Res..

[53] Robinson JT, Thorvaldsdottir H, Wenger AM, Zehir A, Mesirov JP (2017). Variant review with the integrative genomics viewer. Cancer Res..

[54] Thorvaldsdottir H, Robinson JT, Mesirov JP (2013). Integrative Genomics Viewer (IGV): high-performance genomics data visualization and exploration. Brief. Bioinform.

[55] Robinson JT (2011). Integrative genomics viewer. Nat. Biotechnol..

