# Supplementary material for: Potential evidence for transgenerational epigenetic memory in Arabidopsis thaliana following spaceflight
Source: Commun Biol. 2021 Jul 2;4:835. doi: 10.1038/s42003-021-02342-4 (PMC8253727; doi:10.1038/s42003-021-02342-4)
Supplement: Supplementary file 1 — Supplementary Information [file 42003_2021_2342_MOESM1_ESM.pdf]

Supplementary information

Article title: **Potential evidence for transgenerational epigenetic memory in *Arabidopsis thaliana* following spaceflight**

Peipei Xu[1#], Haiying Chen[1#], Jinbo Hu[1], Weiming Cai[1*]

[1]Laboratory of Photosynthesis and Environment, CAS Center for Excellence in Molecular Plant Sciences, Shanghai Institute of Plant Physiology and Ecology, Chinese Academy of Sciences, No. 300 Fenglin Road, Shanghai 200032, China

[*]Corresponding author. Email:wmcai@cemps.ac.cn. ORCID ID: 0000-0002-3818-5499

[#]contributed equally.

Telephone.86-21-54924248

Fax.86-21-54924015

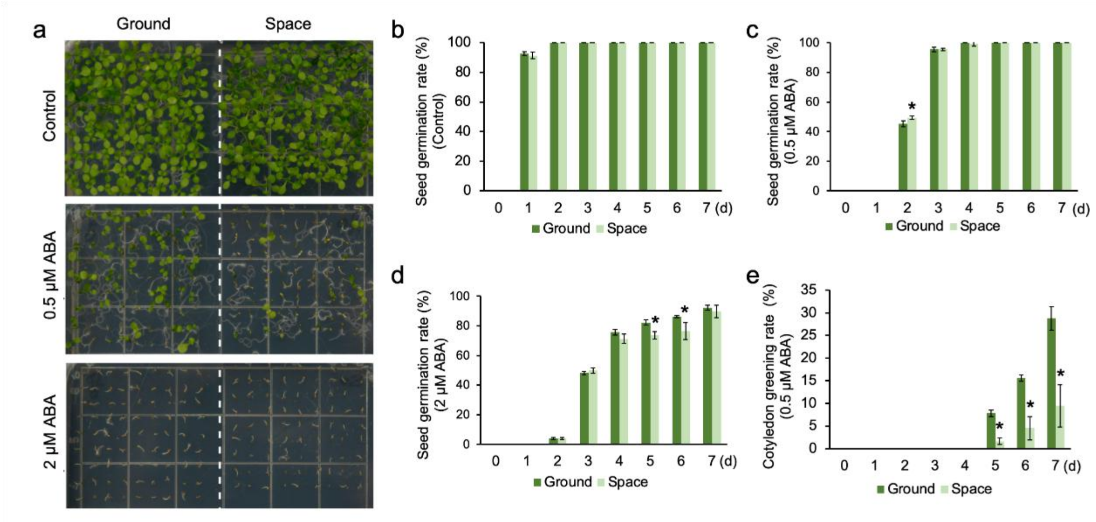

Supplementary figure 1. the ABA sensitivity in the F2 generation of spaceflight microgravity plants and the underground controls

a-e Analysis of germination phenotype and cotyledon greening rate observation under MS media added with various concentration of ABA in the F2 generation of spaceflight and ground control samples. The vertical bars represent the standard deviation with three biological repetition; the asterisk indicates a statistically significant difference between the spaceflight and control samples using the student double-tailed *t* test (p<0.05).

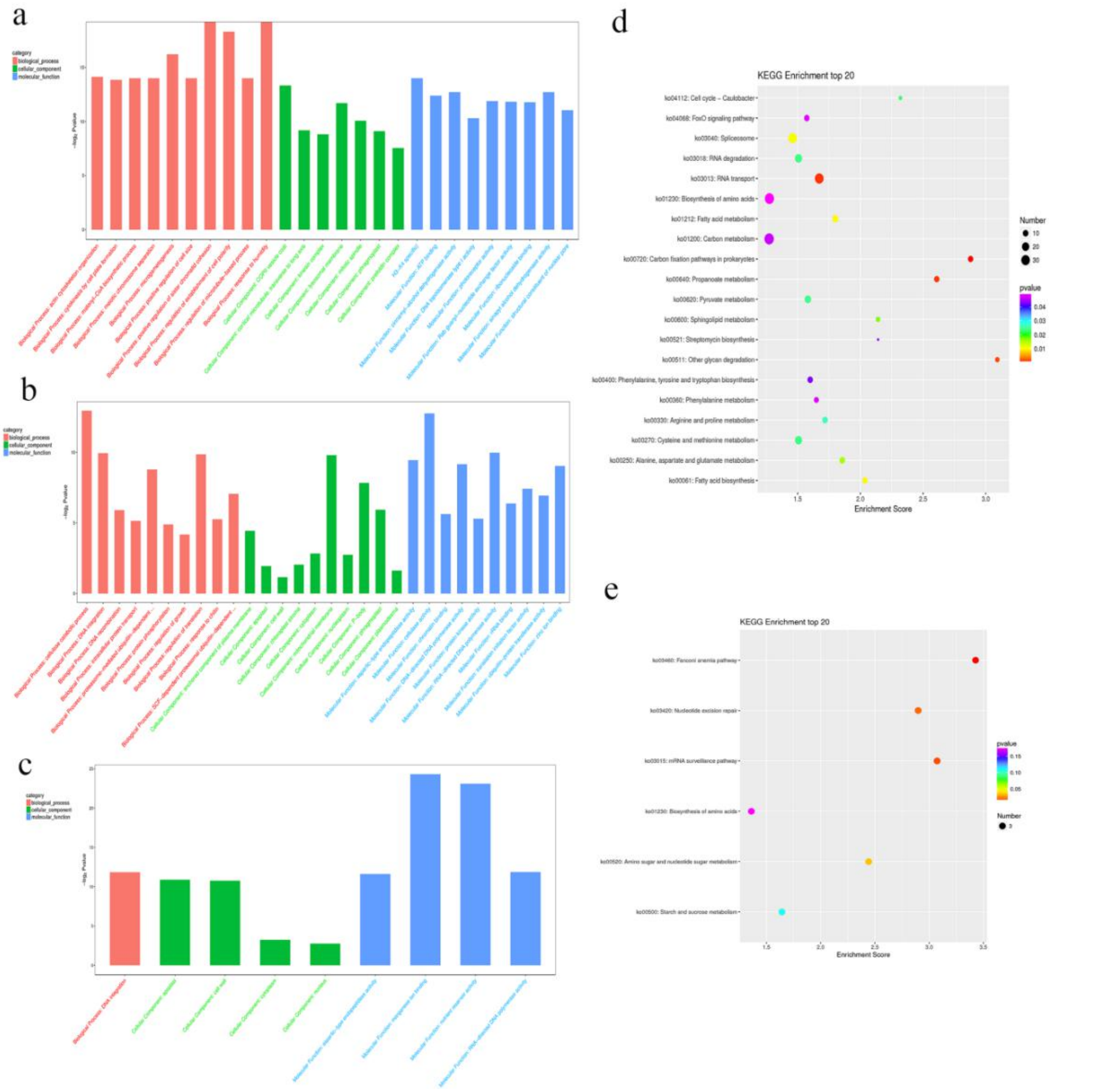

Supplementary figure 2. GO-term enrichment and KEGG pathway analysis of genes with altered methylation levels in the F1 generation Arabidopsis genome

a-c GO-term analysis was carried out on the changes in gene methylation patterns between GO-term enrichment analysis of altered gene methylation patterns in the (**a**) CpG, (**b**) CHG and (**c**) CHH contexts between the F1 generation of control samples and plants under microgravity conditions was conducted. The bar represents the number of genes in the test set belonging to each GO category. The top GO terms are listed ($p<0.05$). **d, e** KEGG pathway enrichment analysis (**d**) CpG, (**e**) CHH differential methylation gene in the Arabidopsis genome between the F1 generation of control samples and plants under microgravity conditions was performed. The size of the circle represents the number of genes, and the color represents the *p*-value.

a F1 DNA methylation patterns

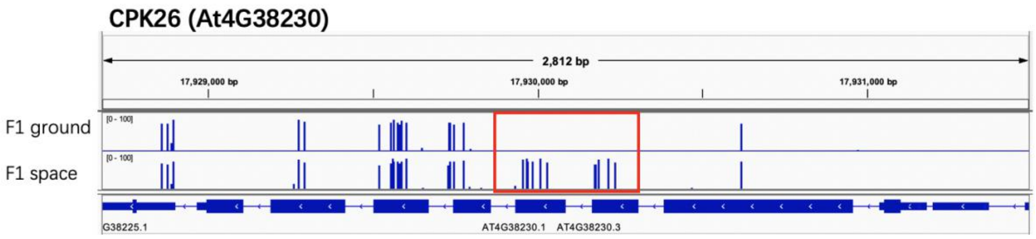

b F2 & F3 CpG island DNA methylation levels

	**1225**	**1243**	**1283**	**1299**	**1327**	**1336**	**1480**	**1499**	**1519**	**1535**	**1545**	**1567**	**Total %**
G2	0	0	8	8	10	10	10	10	10	10	10	0	71.67%
S2	1	0	10	7	10	9	10	10	10	10	10	0	72.5%
G3	0	0	9	9	10	8	10	10	10	10	9	0	70.83%
S3	0	0	10	10	10	10	9	10	8	9	10	0	71.67%

c F2 & F3 Gene expression levels (n=3)

	**Gene expression**	**±SD**	***t*-test**
G2	1	0.1476	0.0829
S2	0.7702	0.09034	
G3	0.4424	0.04861	0.0549
S3	0.5664	0.06347	

Supplementary figure 3. DNA methylation and gene expression levels of *CPK26* (At4g38230) in the offspring

a The distribution of differential methylation of *CPK26* by using the IGV browser graphics in the F1 generation; **b** The methylation level of selected CpG island DNA methylation levels of *CPK26* in the F2 & F3 generations; **c** The gene expression level of *CPK26* in the F2 & F3 generations by using Student's two-tailed *t* test (p<0.05).

a F1 DNA methylation patterns

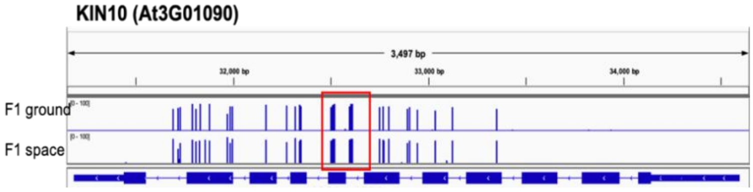

b F2 & F3 CpG island DNA methylation patterns

CpG site	1321	1368	1447	1476	1534	1551	Total %
G2	10	10	0	6	1	10	61.67%
S2	10	9	1	7	1	10	63.33%
G3	10	10	1	9	0	10	66.67%
S2	9	10	1	9	1	9	65%

c F2 & F3 Gene expression level (n=3)

	Gene expression	±SD	t-test
G2	1	0.99538	0.4047
S2	0.9159	0.1241	
G3	0.6628	0.07469	0.119
S3	0.7756	0.06481	

Supplementary figure 4. DNA methylation and gene expression levels of *KIN10* (At3g01090) in the offspring

a The distribution of differential methylation of *KIN10* by using the IGV browser graphics in the F1 generation; **b** The methylation level of selected CpG island DNA methylation levels of *KIN10* in the F2 & F3 generations; **c** The gene expression level of *KIN10* in the F2 & F3 generations by using Student's two-tailed *t* test (p<0.05).

a F1 DNA methylation patterns

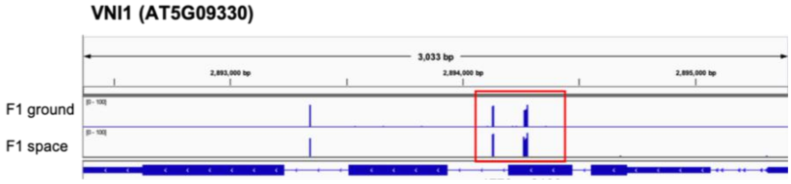

b F2 CpG island DNA methylation levels

	1789	1823	1886	1943	2025	2095	Total %
F2G	0	0	0	1	0	0	1.67%
F2S	0	1	0	0	0	0	1.67%

c F2 & F3 Gene expression levels (n=3)

	Gene expression	±SD	*t*-test
G2	1	0.07465	0.0713
S2	1.154	0.07999	
G3	0.6628	0.0805	0.802
S3	0.7756	0.08414	

Supplementary figure 5. DNA methylation and gene expression levels of *VNI1* (At5g09330) in the offspring

a The distribution of differential methylation of *VNI1* by using the IGV browser graphics in the F1 generation; **b** The methylation level of selected CpG island DNA methylation levels of *VNI1* in the F2 & F3 generations; **c** The gene expression level of *VNI1* in the F2 & F3 generations by using Student's two-tailed *t* test (p<0.05).

a F1 DNA methylation patterns

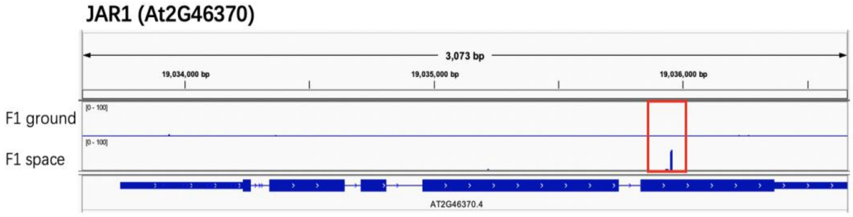

b F2 CpG island DNA methylation levels

	**2407**	**2412**	**2419**	**2429**	**2470**	**2486**	**Total %**
F2G	0	0	0	0	0	0	0%
F2S	0	0	0	0	0	0	0%

c F2 & F3 Gene expression levels (n=3):

	**Gene expression**	**±SD**	***t*-test**
G2	1	0.06672	0.00473
S2	0.7337	0.04624	
G3	0.7974	0.05292	0.0191
S3	0.6552	0.03745	

Supplementary figure 6. DNA methylation and gene expression levels of *JAR1* (At2g46370) in the offspring

a The distribution of differential methylation of *JAR1* by using the IGV browser graphics in the F1 generation; **b** The methylation level of selected CpG island DNA methylation levels of *JAR1* in the F2 & F3 generations; **c** The gene expression level of *JAR1* in the F2 & F3 generations by using Student's two-tailed *t* test ($p<0.05$).

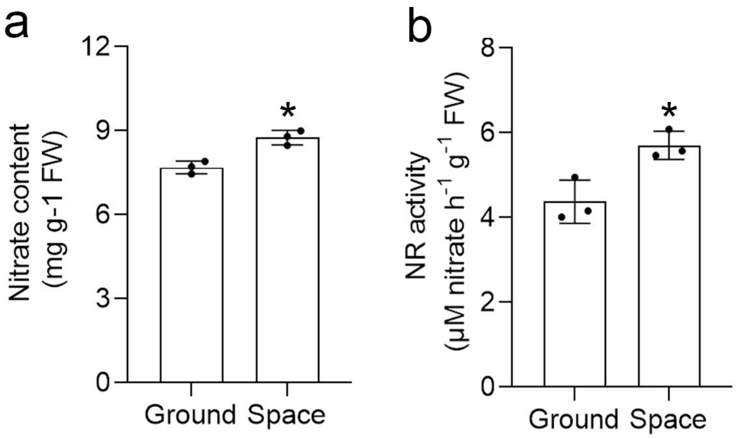

Supplementary figure 7. Nitrate content and NR activity in the F_1 generation of the spaceflight offspring.

a. 50 mg 8-day-old seedlings were used to measure the nitrate content. The prepared samples were ground in liquid nitrogen. Distilled water (1 ml) was applied to the samples, and they were placed in boiling distilled water for 20 min. The mixture was centrifuged at 18 000 g, 4°C, for 10 min. One hundred microliters of the supernatant was placed in a 15-ml Falcon tube, 400 μl of salicylic sulfate acid was added, and the tube was vortexed. Samples were incubated at room temperature for 30 min. The samples were then treated with 9.5 ml of an 8% NaOH solution and cooled at 4°C for 5 min. Nitrate levels were calculated from the measurement of the absorbance at 410 nm. **b.** 6 days old seedlings were used. NR was extracted and measured using an NR Assay Kit (BC0080, SolarBio, Beijing, China). 100 mg samples were extracted and centrifuged at 4000 g for 10 min. The supernatant was analyzed. The absorbance at 520 nm was used to calculate NR activity. Values are given as mean ± SD, n=3. *p<0.05 by Student's *t* test.

Supplementary Table S1. The primers used in BSP analysis

**Transgenes**	**Primers (Sequence 5’-3’)**
CPK26 BSP F:	TTGGATTGAAGGAAATGTTTAAAGTA
CPK26 BSP R:	TCACACAAATCAAATCAAAACAAA
LUHA1 BSP F:	AAAGATTTTGAGAAGAAGAAGTATT
LUHA1 BSP R:	TTCATTAATTAAAAAAAAATAACAATATC
TGA4 BSP F:	TGTTATGAAATATTATTTTTAATTTTTT
TGA4 BSP R:	AAAAAATATAACTTCCTTCACCAAATTT
TGA1 BSP F:	TATTGGGTTGAAGAATAGAATAGAT
TGA1 BSP R:	CATAACAAAATTCACCTAAAAAATA
KIN10 BSP F:	TTTTTGGATTTTAAATGTAATGTAA
KIN10 BSP R:	AAATCTCTAACACCAAAAAAATAAAT
JAR1 BSP F:	TTTTTGGGAGATTTTAGGAGAAAT
JAR1 BSP R:	CACAAAATTTAAAAAACCTTAACA

Supplementary Table S2. Gene-specific primers used in the qRT-PCR experiments

**Genes**	**Primers (Sequence 5'-3')**
CPK26 RT Fl:	AGAGTCTCTCGGAGGAAGAAA
CPK26 RT R:	CTTTCAGCTCGTCAAACGTAATC
LUHA1 RT F:	CAGTCTCCCACATTCACACTT
LUHA1 RT R:	GACGCCATCGTTTCTCTCTATC
TGA4 RT F:	TCTTGTGGATAGGCGGATTTAG
TGA4 RT R:	GTTGACATGATTGCCTCAGATTAC
TGA1 RT F:	GAAATCGTCTGCTGCCAAAG
TGA1 RT R:	CGAAATCCGCCAATCCATAAG
JAR1 RT F:	CGGCTTGGAGATGTGGTAAA
JAR1 RT R:	AGGTCTCTTTCGGTGTTCTTG
KIN10 RT F:	TGTGAACTCTGGTGAGCTATTT
KIN10 RT R:	GGCAGTATTCCACTCCTGATATT
ACTIN2 RT F:	TGTGCCAATCTACGAGGGTTT
ACTIN2 RT R:	ATTTCTTTGCTCATACGGTCAG
