# Supplementary material for: Potential evidence for transgenerational epigenetic memory in Arabidopsis thaliana following spaceflight
Source: Commun Biol. 2021 Jul 2;4:835. doi: 10.1038/s42003-021-02342-4 (PMC8253727; doi:10.1038/s42003-021-02342-4)
Supplement: Supplementary file 3 — Reporting Summary [file 42003_2021_2342_MOESM3_ESM.pdf]

nature research

Corresponding author(s): Weiming Cai

Last updated by author(s): Apr 9, 2021

Reporting Summary

Nature Research wishes to improve the reproducibility of the work that we publish. This form provides structure for consistency and transparency in reporting. For further information on Nature Research policies, see our Editorial Policies and the Editorial Policy Checklist.

Statistics

For all statistical analyses, confirm that the following items are present in the figure legend, table legend, main text, or Methods section.

n/a	Confirmed	
☐	☒	The exact sample size (*n*) for each experimental group/condition, given as a discrete number and unit of measurement
☐	☒	A statement on whether measurements were taken from distinct samples or whether the same sample was measured repeatedly
☐	☒	The statistical test(s) used AND whether they are one- or two-sided<br>*Only common tests should be described solely by name; describe more complex techniques in the Methods section.*
☐	☒	A description of all covariates tested
☐	☒	A description of any assumptions or corrections, such as tests of normality and adjustment for multiple comparisons
☐	☒	A full description of the statistical parameters including central tendency (e.g. means) or other basic estimates (e.g. regression coefficient) AND variation (e.g. standard deviation) or associated estimates of uncertainty (e.g. confidence intervals)
☒	☐	For null hypothesis testing, the test statistic (e.g. *F*, *t*, *r*) with confidence intervals, effect sizes, degrees of freedom and *P* value noted<br>*Give P values as exact values whenever suitable.*
☒	☐	For Bayesian analysis, information on the choice of priors and Markov chain Monte Carlo settings
☒	☐	For hierarchical and complex designs, identification of the appropriate level for tests and full reporting of outcomes
☒	☐	Estimates of effect sizes (e.g. Cohen's *d*, Pearson's *r*), indicating how they were calculated

Our web collection on statistics for biologists contains articles on many of the points above.

Software and code

Policy information about availability of computer code

Data collection	Genetic ontology (GO) annotations were performed using agriGO v2.051,52 (http:://systemsbiology. cau.edu.cn/agriGOv2/index.php), a public online tool and database. The significance level for GO terms was FDR<0.05. KEGG (http://www.genome.jp/kegg/) analysis was used to determine the pathway enrichment of functional DMRs or differential expression genes with p values less than 0.05.A repeat masking program (http://www.repeatmasker.org/) was used to separate and analyze TEs. We used Fisher's exact tests to study TEs for changes in methylation levels. The FDR method was used multiple times to test adjustments. A p value less than 0.05 was defined as a methylated TEs with significant changes. The Integrated Genome Viewer (IGV) (http://software.broadinstitute.org/software/igv/) was used to visualize methylation levels of the CpG, CHG, and CHH contexts of chromosomes.
Data analysis	Go Enrichment analysis, KEGG analysis, repeat masking program analysis,

For manuscripts utilizing custom algorithms or software that are central to the research but not yet described in published literature, software must be made available to editors and reviewers. We strongly encourage code deposition in a community repository (e.g. GitHub). See the Nature Research guidelines for submitting code & software for further information.

Data

Policy information about availability of data

All manuscripts must include a data availability statement. This statement should provide the following information, where applicable:

- Accession codes, unique identifiers, or web links for publicly available datasets
- A list of figures that have associated raw data
- A description of any restrictions on data availability

Data related to the article can be obtained under reasonable request

Field-specific reporting

Please select the one below that is the best fit for your research. If you are not sure, read the appropriate sections before making your selection.

- [x] Life sciences
- [ ] Behavioural & social sciences
- [ ] Ecological, evolutionary & environmental sciences

For a reference copy of the document with all sections, see nature.com/documents/nr-reporting-summary-flat.pdf

Life sciences study design

All studies must disclose on these points even when the disclosure is negative.

Sample size	the sample number of the root length and flowering time of F1 generation exceeded 40 individuals, and the sample number of F2 and F3 generation exceeded 100 individuals. The flowering time analysis of the three successive F1, F2 and F3 generations of spaceflight and ground control samples (n>30 for each generation). The experiment was conducted in the F1 generation and the F2/F3 generations of Arabidopsis seedlings at 8-d-old and 7-d-old, respectively. The data shown are the average values of 14 samples in the F1 generation (n = 14) and 24 samples in the F2/F3 generation.
Data exclusions	No data were excluded
Replication	Three repeats were performed
Randomization	The samples in each group were randomly selected and separately measured
Blinding	The investigators were blinded to group allocation during data collection and analysis

Reporting for specific materials, systems and methods

We require information from authors about some types of materials, experimental systems and methods used in many studies. Here, indicate whether each material, system or method listed is relevant to your study. If you are not sure if a list item applies to your research, read the appropriate section before selecting a response.

Materials & experimental systems

n/a	Involved in the study	
x		Antibodies
x		Eukaryotic cell lines
x		Palaeontology and archaeology
x		Animals and other organisms
x		Human research participants
x		Clinical data
x		Dual use research of concern

Methods

n/a	Involved in the study	
x		ChIP-seq
x		Flow cytometry
x		MRI-based neuroimaging

April 2020
